# Effects of Acorns on Meat Quality and Lipid Metabolism-Related Gene Expression in Muscle Tissues of Yuxi Black Pigs

**DOI:** 10.3390/metabo14110578

**Published:** 2024-10-26

**Authors:** Zhe Sun, Yadi Chang, Luyao Huang, Siyuan An, Dongyang Liu, Jinzhou Zhang, Zhiguo Miao

**Affiliations:** College of Animal Science and Veterinary Medicine, Henan Institute of Science and Technology, Xinxiang 453003, China; proyts2017@gmail.com (Z.S.); bcd679601@gmail.com (Y.C.); acdd8088@gmail.com (L.H.); 6bing7@gmail.com (S.A.); pddedg2@gmail.com (D.L.); forthegodsake22@gmail.com (J.Z.)

**Keywords:** acorn, Yuxi black pigs, intramuscular fat, fatty acids

## Abstract

Objectives: The aim of this study was to investigate the effects of acorn diets on the composition of fatty acid (FA) and the intramuscular fat (IMF) content in Yuxi black pigs. Methods: Ninety Yuxi black pigs with similar body weight (99.60 ± 2.32 kg) were randomly divided into five groups. The control group was fed a basal diet, and the AD20, AD30, AD40, and AD50 groups were fed experimental diets which contained 20%, 30%, 40%, and 50% acorns, respectively. The feeding experiment lasted for 120 days. Results: The results showed that compared with the control group, the content of SFA in *longissimus dorsi* and *biceps femoris* tissues in the AD30 group decreased by 8.57% and 20.10%, and the content of MUFA increased by 5.40% and 15.83%, respectively, while the PUFA content of *biceps femoris* increased by 5.40% (*p* < 0.05). Meanwhile, the IMF content of the AD30 group was significantly higher than that of the control group in the *longissimus dorsi* and *biceps femoris*. In addition, the mRNA expression levels of the *ATGL*, *PPARγ*, and *FABP4* genes in *longissimus dorsi* (*p* < 0.05) were up-regulated, and *HSL* were down-regulated (*p* < 0.05) in the AD30 group. In the *biceps femoris* of the AD30 group, it was observed that the expression levels of the *ACC* and *FAS* genes were up-regulated (*p* < 0.05), while *HSL* and *ATGL* genes were down-regulated (*p* < 0.05). Conclusions: These results demonstrated that the addition of appropriate amounts of acorn to the diet (a 30% acorn diet) could improve the nutritional value of pork.

## 1. Introduction

In modern society, people are paying more attention to meat quality and human health. The quality of pork is closely related to human health. Previous studies have shown that the intramuscular fat (IMF) content and fatty acid (FA) composition are essential determining factors of the nutritional value of pork [1]. Intramuscular fat (IMF) is the fat between muscle fibers or muscle bundles and its greatest deposition is in the later stages of the growth process [2]. Its content has a direct effect on the flavor, juiciness, and tenderness of meat [3]. Several factors such as diet, breed, age, gender, and genetics influence the IMF content of porcine carcasses [4]. Additionally, IMF is regulated and controlled by multiple genes, such as the acetyl-coa carboxylase (*ACC*), fatty acid synthase (*FAS*), hormone-sensitive lipase (*HSL*), adipose triglyceride lipase (*ATGL*), peroxisome proliferator-activated receptor gamma (*PPARγ*), and adipose fatty acid binding protein 4 (*FABP4*) genes [5,6,7]. Han’s study found that adipose triacyl glyceride lipase, carnitine palmitoyl transferase 1B, and fatty acid-binding protein 4 were up-regulated by the consumption of Eumoides leaves extract. The down-regulation of CCAAT/enhancer-binding protein A, fatty acid translocase, carnitine palmitoyl transferase 1B, and adipose triacyl glyceride lipase thereby regulates the lipid metabolism of pigs, improving pork quality [8]. IMF contains a significant amount of unsaturated fatty acids (PUFAs), mainly oleic acid (18:1cis-9), linoleic acid (18:2n-6), and α-linolenic acid (18:3n-3) [9]. Therefore, the IMF content can promote human health by improving the quality of meat from animals [10].

In some developed countries, people are encouraged to reduce their consumption of SFA and increase their intake of PUFA to maintain their health and reduce the incidence of metabolic diseases [11]. In China, people’s view of the consumption of pork products has undergone a shift from quantity to quality. The meat and nutritional quality of pork are usually influenced by various factors such as nutritional levels [12], environmental factors [13], and genetic resources [14,15]. Our previous research also found that nutritional and genetic factors could significantly affect the quality of pork [7,16,17], which indicated that the characteristics and quality of pork can be regulated and controlled by dietary/nutritional factors.

Acorns are the fruit of oak trees, a group which consists of approximately 300 species in China. They have a high nutritional value and are characterized by high starch, crude fat, and PUFA contents, and their nutritional value and calories are similar to corn and sorghum [18,19]. They are usually consumed in health products such as acorn noodles, acorn biscuits, and acorn bean jelly in China [17]. As the nut of oak trees, acorns have the advantage of a high yield and low price. For this reason, they can be used as a safe wild raw material resource for animal feed production. Growing studies have found that dietary acorn supplementation could improve the meat quality of pigs and regulate the percentage of fatty acids in pork [20,21]. A previous study found that the content of monounsaturated fatty acids (especially oleic acid) in the subcutaneous adipose tissue of Iberian ham was significantly increased by dietary acorn supplementation [22]. In addition, under free-range conditions (feeding on acorns and grass), the IMF content in the muscular tissues was increased, which improved the meat quality of Iberian pigs [23]. These results suggest that a diet including acorns has a positive effect on both pork quality and human health. But these studies did not to regulate the fatty acid composition and lipid metabolism-related gene expression level for further study. Therefore, it was hypothesized that acorns could regulate fat deposition by affecting the expression of genes related to lipid metabolism, thereby improving the meat quality characteristics of pigs.

The Yuxi black pig is a famous local breed which is distributed primarily in the mountainous regions of the west of Henan province, China (such as Luanchuan County and Lushi County). These pigs are characterized by early maturation, strong immune ability, a tolerance to roughage, and excellent meat quality [24]. Another study found that Yuxi black pigs have better carcass traits and excellent meat quality, with an abundant fatty acid content in muscle and an appropriate ratio of unsaturated to saturated fatty acids [25]. In addition, the Yuxi black pig is also characterized by slow growth efficiency, thicker backfat thickness, and longer feeding periods. At present, the effects of an acorn diet on the fatty acid and molecular mechanisms of lipid metabolism in Yuxi black pigs have not been found. Therefore, this study was the first to investigate the effects of acorns on IMF content and FA compositions and the expression levels of lipid metabolism-related genes in the muscular tissues of Yuxi black pigs. These data will provide a scientific basis for the development and utilization of acorns as a new type of feed resource for pigs.

## 2. Materials and Methods

### 2.1. Experimental Design and Diets

The Animal Care and Use Committee of Henan Institute Science and Technology (Xinxiang, China) approved all pig handling protocols in this study. Ninety Yuxi black pigs (sex-balanced) with a similar body weight (99.60 ± 2.32 kg) were randomly divided into five groups (all experimental pigs were weighed and grouped according to their weight so that the mean weight of the experimental pigs in each group was similar): one control group and four acorn-diet experimental groups. A basal diet was fed to the control group, and the AD20, AD30, AD40, and AD50 groups were fed experimental diets which contained 20%, 30%, 40%, and 50% acorns, respectively. There were three replicates, with six pigs per replicate, of each group, and the feeding experiment lasted for 120 days. During the feeding period of this batch of test animals, the pen size was 20 square meters, the fence was a fixed fence, the floor was a manure leakage floor, the temperature was about 24 °C, and there was natural ventilation. All diets used in this study were formulated based on the National Research Council (NRC 1998) recommendation, with slight modification fattening pigs; the ratio of crude protein (CP) to metabolizable energy (ME) was similar in all experimental diets. (In the diet configuration process, since energy and crude protein could not be equal, we adjusted the protein–energy ratio to be nearly balanced. The increase in acorn content affected the level of energy and protein in the diet, and the regulation of this change was partially supplemented by wheat bran and soybean meal.) The composition of the diets used in this experiment is shown in Table 1. The results of the fatty acid composition of the diets determined for the different groups are shown in Table 2. All experimental pigs were fed twice per day at 6 o’clock and 18 o’clock, respectively. During this period, all pigs had ad libitum access to water via a nipple drinker, and all pigs received an average daily consumption of 2.5 kg of the experimental diet to control their body weight.

### 2.2. Slaughter and Sample Collection

After the feeding experiment, a total of 30 experimental pigs (6 pigs from each group and 2 pigs per replicate, gender-balanced), based on their similar average body weight, were selected, weighed, and slaughtered (the pigs were electroshocked, exsanguinated, and dissected) to collect experimental samples after they had fasted for 12 h. Their *longissimus dorsi* (*longissimus dorsi* were taken from between the third- and fourth-last ribs) and *biceps femoris* (*biceps femoris* was harvested from the posterior side of the femur) were collected in 1.5 mL EP tubes and rapidly frozen in liquid nitrogen; all samples were transferred to an ultra-low-temperature freezer (Jin Youning Instrument Co., Ltd., Zhengzhou, China) at −80 °C for storage until sample analysis.

### 2.3. Intramuscular Fat Content and FA Composition Analysis

The IMF content of *longissimus dorsi* and *biceps femoris* were analyzed by the Soxhlet extraction method [26]. Their FA composition was determined using previously reported methods based on FA methyl ester synthesis [22,27]. The muscle tissue was lyophilized, and the lipids were extracted from the *longissimus dorsi* and *biceps femoris* by the one-step methyl ester method; the extracted lipid was hydrolyzed in 2 mL of KOH–methanol (C = 0.5 mol/L) with shaking for 1 min. The mixture was reacted in water at 95 °C with continual shaking for a period of 10 min. The free fatty acid mixture was subjected to esterification in a 2 mL solution of BF3–methanol (W = 10%) with shaking for 10 s. The mixture was subjected to a 20 min reaction at 80 °C in a water bath with continuous agitation. Subsequently, 1 mL of n-hexane and 5 mL of a saturated NaCl solution were introduced into the mixture. Following a 60 s shaking period, the mixture was subjected to centrifugation at 3000 rpm for a further 15 min. Thereafter, 800 μL of the resulting fatty acid methyl esters were collected and analyzed by means of gas chromatography (Agilent-7890, Agilent Technologies Inc., Santa Clara, CA, USA). The data were presented as a percentage of the total *FAS* identified, and the saturated fatty acid (SFA), monounsaturated fatty acid (MUFA), polyunsaturated fatty acids (PUFAs), PUFA/SFA ratio, and n6/n3 PUFA ratio were calculated, respectively.

### 2.4. Nutritional Indexes

Based on the data of the FAS composition in *longissimus dorsi* and *biceps femoris*, the atherogenicity index (AI), thrombogenicity index (TI), fatty acid unsaturation index (UI), peroxidation trend index (PI), Health-Promoting index (HPI), nutrition value index (NVI), hypocholesterolemic/hypercholesterolemic ratio (h/H), and DHA (docosahexaenoic acid, C22:6 n-3) + EPA (eicosapentaenoic acid, C20:5 n-3) were further calculated according to equations introduced in previous studies [21,28].

### 2.5. Quantitative Real-Time PCR Analysis

Total RNA was extracted from the *longissimus dorsi* and *biceps femoris* of Yuxi black pigs using TRIzol reagent (Takara Bio Inc., Tokyo, Japan). The percentage and purity of the extracted RNA were measured using a spectrophotometer (IMPLEN, Westlake Village, CA, USA), and the optical density value of the extracted RNA was 1.8 ≤ 260/280 ≤ 2.0. Using the total RNA (approximately 1 μg) of every sample, complementary DNA (cDNA) was synthesized via the PrimeScript^TM^ RT Reagent Kit (Takara Bio Inc., Tokyo, Japan). RT-PCR was carried out using a Bio-Rad SYBR green RT-PCR kit (Thermo Fisher Scientific, Hercules, CA, USA) and ViiA^TM^ 7 real-time PCR system. The primer sequences utilized in this experiment were designed based on the known sequences in GenBank (Table 3). The expression levels of lipid metabolism-related genes were measured by qRT-PCR, with GAPDH as an internal reference; the expression levels were calculated using the 2^−ΔΔCt^ method.

### 2.6. Statistical Analysis

The data in this study were analyzed using a one-way analysis of variance (ANOVA) followed by Tukey’s multiple comparison tests on SPSS26.0 (SPSS Inc., Chicago, IL, USA) software. GraphPad Prism 6 software (GraphPad, San Diego, CA, USA) was used for data visualization. The results were presented as means ± SEM, and significance was declared at *p* < 0.05. The bivariate correlation procedure of SPSS 26.0 was utilized to calculate the correlation between IMF content and lipid metabolism-related gene expression.

## 3. Results

### 3.1. Growth Performance

**Figure 1:**
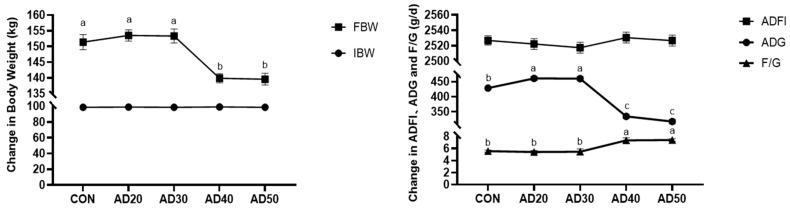
Effects of acorn diets on the growth performance of Yuxi black pigs IBW: initial weight; FBW: final weight; ADG: average daily gain; ADFI: average daily feed intake; F/G: ratio of feed to body weight; CON: diet containing 0% acorns; AD20: diet containing 20% acorns; AD30: 30% acorn diet; AD40: 40% acorn diet; AD50: 50% acorn diet. The values within a row marked with a different superscript are significantly different (*p* < 0.05).

As shown in Figure 1, compared with the control group, the ADG in the AD20 and AD30 groups was significantly increased and the FBW and ADG in the AD40 and AD50 groups were significantly decreased, while F/G was significantly increased (*p* < 0.05). There were no significant differences in IBW or ADFI among all groups (*p* > 0.05).

### 3.2. IMF Content

**Figure 2:**
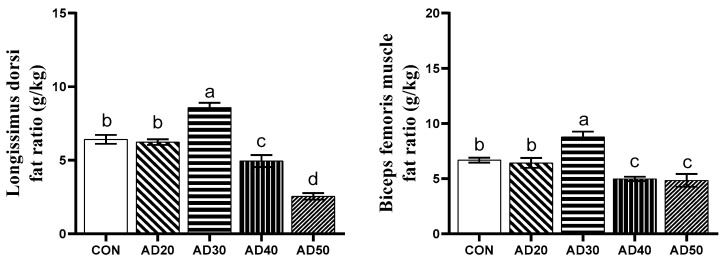
Effect of acorn diets on the intramuscular fat content in *longissimus dorsi* and *biceps femoris* of Yuxi black pigs. CON: diet containing 0% acorns; AD20: diet containing 20% acorns; AD30: 30% acorn diet; AD40: 40% acorn diet; AD50: 50% acorn diet. The values are presented as mean values with their standard errors shown as vertical bars. The values within a row marked with a different superscript are significantly different (*p* < 0.05).

The effects of acorn diets on the IMF content in the muscle tissues of Yuxi black pigs are shown in Figure 2. The IMF content in *longissimus dorsi* and *biceps femoris* in the AD30 group was significantly higher than that in the control group, by 33.64% and 31.44% (*p* < 0.05), whereas, compared with the control group, the AD40 and AD50 groups had a lower IMF content in their *longissimus dorsi* and *biceps femoris* by 22.90%, 60.42%, 25.45%, and 27.40% (*p* < 0.05). No significant differences in IMF content were observed between the control group and the AD20 group (*p* > 0.05).

### 3.3. Fatty Acid Composition

#### 3.3.1. Fatty Acid Composition in *longissimus dorsi*

The decrease in SFA and increase in MUFA and PUFA are important for improving pork quality and promoting human health [29]. As shown in Table 4, compared with the control group, the AD30 group had a lower total SFAS percentage and higher total MUFAS percentage in its *longissimus dorsi* (*p* < 0.05). Meanwhile, the AD40 group had higher total PUFA levels in its *longissimus dorsi* than the control group (*p* < 0.05). Compared with the control group, the percentage of C16:0 in the AD40 and AD50 groups was significantly decreased, and the percentage of C17:0 in the AD20 and AD40 groups was significantly increased (*p* < 0.05); the C20:0 percentage in the AD40 group and the C22:0 percentage in the AD20 group were significantly increased (*p* < 0.05). At the same time, the AD20 and AD50 groups’ C18:0 percentage was significantly higher than that of control group, while the AD30 and AD40 groups’ C18:0 percentage was significantly lower than that of the control group (*p* < 0.05). Furthermore, the percentages of C10:0, C12:0, C14:0, C15:0, and C23:0 in each experimental group were not significantly different from those in the control group (*p* > 0.05). In a comparison of the test groups and control group, each group’s C16:1 percentage was significantly reduced, and the percentage of C17:1 in the AD30, AD40, and AD50 groups was significantly increased (*p* < 0.05). The percentage of C18:1 was significantly increased in the AD30 group but decreased in the AD20, AD40, and AD50 groups (*p* < 0.05). Moreover, the percentage of C14:1, C15:1, C20:1, and C22:1 in each experimental group was not significantly different from that in the control group (*p* > 0.05). The increase in C18:2n-6 and C18:3n-3 had a positive effect on lowering blood lipids and blood pressure [30]. Compared with the controls, the percentage of C18:2 n-6 and C18:3 n-3 in each experimental group was significantly increased (*p* < 0.05). The percentage of C18:3 n-6 in the AD20 group was significantly increased (*p* < 0.05). The percentage of C20:3 n-6 and C20:3 n-3 was significantly increased in the AD20 and AD40 groups (*p* < 0.05). Meanwhile, C20:2 n-6 in the AD30 and AD40 groups was significantly higher than that in control group *(p* < 0.05). In addition, the percentages of C20:4 n-6, C20:5 n-3, and C22:2 n-6 in each experimental group were not significantly different from those in the control group (*p* > 0.05).

#### 3.3.2. Fatty Acid Composition in *biceps femoris*

As shown in Table 5, compared with the control group, the AD30 group had lower total SFAS and total PUFAS levels and higher total MUFAS levels in their *biceps femoris* (*p* < 0.05). Meanwhile, the AD40 group had lower total PUFA levels than the control, AD20, AD30, and AD50 groups (*p* < 0.05). Compared with the controls, each groups’ C12:0 percentage significantly reduced; the percentage of C14:0, C17:0, and C18:0 in the AD20, AD30, and AD50 groups was significantly decreased (*p* < 0.05). The percentage of C15:0 in the AD40 and AD50 groups was significantly decreased (*p* < 0.05). The AD20 and AD30 groups’ C20:0 percentage decreased significantly (*p* < 0.05). The C22:0 in the AD20, AD40, and AD50 groups was significantly decreased (*p* < 0.05). At the same time, the AD40 group’s C16:0 percentage was significantly higher than that of the control group and the AD20 and AD30 groups’ C16:0 percentage was significantly lower than that of the control group (*p* < 0.05). Furthermore, the percentage of C10:0 and C23:0 in each experimental group was not significantly different from that in the control group (*p* > 0.05). From a comparison of the test groups and control group, the percentage of C14:1 in the AD20 and AD30 groups was significantly decreased (*p* < 0.05); the percentage of C15:1 in the AD20, AD40, and AD50 groups was significantly decreased (*p* < 0.05); the C16:1 in the AD30, AD40, and AD50 groups increased significantly (*p* < 0.05); and the AD40 and AD50 groups’ C22:1 percentage decreased significantly (*p* < 0.05). The percentage of C17:1 in the AD40 group was significantly higher than that in control group (*p* < 0.05). Meanwhile, the percentage of C18:1 and C20:1 in each experimental group was significantly higher than that in the control group (*p* < 0.05). Compared with the control group, the percentage of C18:2 n-6 in each experimental group was significantly decreased (*p* < 0.05). The AD30 group’s C18:3 n-6 percentage significantly reduced (*p* < 0.05). The percentage of C20:3 n-6, C20:3 n-3, C20:4 n-6, and C20:5 n-3 in the AD40 and AD50 groups was significantly decreased (*p* < 0.05). The AD30 group’s C18:3 n-3, C20:3 n-6, C20:3 n-3, C20:5 n-3, and C22:2 n-6 percentages increased significantly (*p* < 0.05). At the same time, the AD20, AD40, and AD50 groups’ C18:3 n-3 and C22:2 n-6 percentages were significantly lower than those of the control group (*p* < 0.05). The C20:2 n-6 percentage was significantly lower in the AD30 and AD40 groups and significantly higher in the AD50 group compared to the control group (*p* < 0.05). In addition, the percentage of C22:6 n-3 in each experimental group was not significantly different from that in the control group (*p* > 0.05).

### 3.4. Nutritional Indicators of Fatty Acid Composition

#### 3.4.1. Nutritional Indicators of Fatty Acid Composition in *longissimus dorsi*

As shown in Table 6, compared with the control group, the ratio of n-6: n-3 in the *longissimus dorsi* of the AD20 and AD40 groups was lower, while that of the AD30 group was higher (*p* < 0.05). Meanwhile, compared with the control group, the AD20 and AD40 groups had higher EPA+DHA and PI values, and the AD30 group had lower TI values (*p* < 0.05). While all experimental groups (AD20, AD30, AD40, and AD50) had higher PUFA: SFA and h/H ratios (*p* < 0.05), the AD40 and AD50 groups had higher AI values (*p* < 0.05), the AD40 group had a higher UI value (*p* < 0.05), and the AD20 and AD50 groups had higher NVI values (*p* < 0.05). However, there were no significant differences in LA/ALA ratios or HPI values among all groups (*p* > 0.05).

#### 3.4.2. Nutritional Indicators of Fatty Acid Composition in *biceps femoris*

As shown in Table 7, compared with the control group, n-6:n-3 and LA/ALA were significantly decreased and PUFA: SFA and UI were significantly increased in the AD30 group, while EPA+DHA and UI were significantly decreased in the AD40 and AD50 groups (*p* < 0.05). Meanwhile, the AI and TI of the AD20, AD30, and AD50 groups were significantly lower than those of the control group, while their HPI, NVI, and h/H were significantly higher than those of the control group (*p* < 0.05). While the AD40 group’s AI and TI were higher, its HPI and h/H were lower (*p* < 0.05). In addition, the PUFA: SFA and PI of the AD20, AD40, and AD50 groups were significantly lower than those of the control group (*p* < 0.05). The AI and TI of the AD20, AD30, and AD50 groups were significantly lower than those of the control group (*p* < 0.05).

### 3.5. Intramuscular Fat-Related Gene Expression

#### 3.5.1. In *longissimus dorsi*

**Figure 3:**
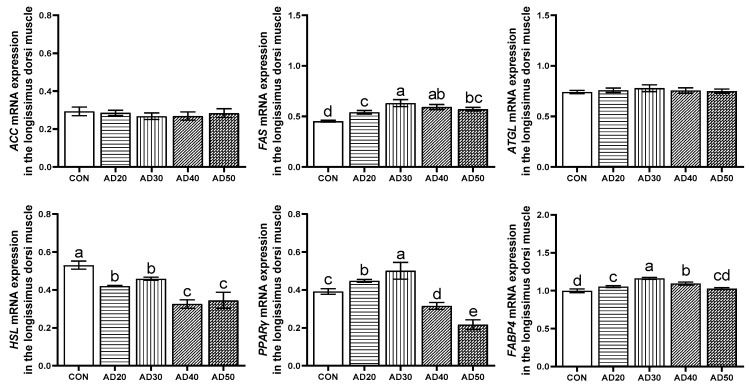
Expression of the fat metabolism-related genes *ACC*, *FAS*, *ATGL*, *HSL*, *PPARγ*, and *FABP4* in the *longissimus dorsi* of Yuxi black pigs. *ACC*, acetyl-coa carboxylase; *FAS*, fatty acidsynthase; *HSL*, hormone-sensitive lipase; *ATGL*, adipose triacylglyceride lipase; *PPARγ*, peroxisome proliferator-activated receptor γ; *FABP4*, fatty acid transport protein 4. CON: diet containing 0% acorn; AD20: diet containing 20% acorns; AD30: 30% acorn diet; AD40: 40% acorn diet; AD50: 50% acorn diet. The values are presented as mean values with their standard deviation shown as vertical bars. The same lowercase letters indicate non-significant differences (*p* > 0.05), while different lowercase letters indicate significant differences (*p* < 0.05).

The effects of adding acorn to diets on the expression of genes related to lipid metabolism in the *longissimus dorsi* of Yuxi black pigs are shown in Figure 3. *ACC* and *ATGL* mRNA expression levels were not significantly different among any of the groups (*p* > 0.05). Compared with the control group, all experimental groups had higher *FAS* mRNA levels (*p* < 0.05); the AD20 and AD30 groups had higher *PPARγ* mRNA levels (*p* < 0.05), while the AD20, AD30, and AD40 groups had higher *FABP4* mRNA levels (*p* < 0.05), and all groups had lower *HSL* mRNA levels (*p* < 0.05). In addition, the AD30 group had the highest expression levels of the *FAS*, *PPARγ*, and *FABP4* genes in their *longissimus dorsi*.

#### 3.5.2. In the *biceps femoris*

**Figure 4:**
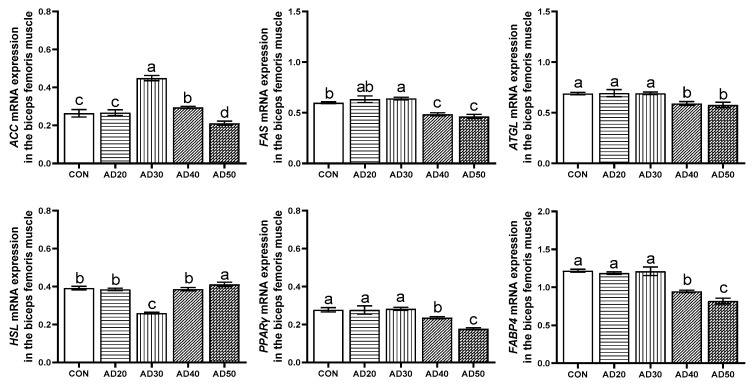
Expression of the fat metabolism-related genes *ACC*, *FAS*, *ATGL*, *HSL*, *PPARγ*, and *FABP4* in the *biceps femoris* of Yuxi black pigs. *ACC*, acetyl-coa carboxylase; *FAS*, fatty acidsynthase; *HSL*, hormone-sensitive lipase; *ATGL*, adipose triacylglyceride lipase; *PPARγ*, peroxisome proliferator-activated receptor γ; *FABP4*, fatty acid transport protein 4. CON: diet containing 0% acorns; AD20: diet containing 20% acorns; AD30: 30% acorn diet; AD40: 40% acorn diet; AD50: 50% acorn diet. The values are presented as mean values with their standard deviation shown as vertical bars. The same lowercase letters indicate non-significant differences (*p* > 0.05), while different lowercase letters indicate significant differences (*p* < 0.05).

As is shown in Figure 4, the ACC mRNA expression in the AD30 and AD40 groups was significantly higher than that in the control group, while that in the AD50 group was significantly lower than that in the control group (*p* < 0.05). At the same time, compared with the control group, the expression level of FAS mRNA in the AD30 group was significantly increased, while that in the AD40 and AD50 groups was significantly decreased (*p* < 0.05). The HSL mRNA expression level was significantly decreased in the AD30 group and significantly increased in the AD50 group (*p* < 0.05). In addition, ATGL, PPARγ, and FABP4 mRNA expression levels were not significantly different in the AD20 and AD30 groups compared with the control group (*p* > 0.05) but were significantly decreased in the AD40 and AD50 groups (*p* < 0.05).

### 3.6. Correlation Between Lipid Metabolism-Related Gene Expression and IMF Content

As shown in Table 8, in the *longissimus dorsi* of Yuxi black pigs, the mRNA levels of *PPARγ* and *FABP4* were positively correlated with the IMF content (*p* < 0.05), while the *HSL* mRNA level was negatively correlated with IMF content (*p* < 0.05). In the *biceps femoris*, the *ACC*, *FAS*, *PPARγ*, and *FABP4* mRNA levels were positively correlated with IMF content, and the *ATGL* and *HSL* mRNA levels were negatively correlated with IMF content (*p* < 0.05).

## 4. Discussion

### 4.1. Growth Performance

The growth performance of an animal is an important indicator in evaluating its breeding level. In intensive production, people mainly judge whether pigs grow healthily by performance indicators such as their weight or daily gain. In this study, it was found that the ADG of pigs could be significantly increased by feeding them 20% and 30% acorn diets. It has been found that the addition of acorns to the diet can improve the ADG of Iberian black pigs [22]. Another study found that feeding an acorn diet significantly improved the growth performance and pork quality of pigs [20]. This is consistent with the results of the present study, indicating that adding an appropriate amount of acorn to the diet can improve the growth performance of pigs.

### 4.2. IMF Content

The IMF content and its FAS composition are closely related to the tenderness and flavor of pork, and health concerns [31]. Improving the eating quality and nutritional value of pork by regulating IMF deposition and its FA characteristics is a scientific research hotspot for researchers [32]. Pork is the main source of dietary FA intake for humans, which is closely related to human health [33]. Previous researchers have provided some strategies to significantly increase the IMF content in pork without increasing subcutaneous fat, thereby improving pork quality [34]. Our study found that feeding a diet supplemented with acorns significantly increased the IMF content in the *longissimus dorsi* and *biceps femoris* of the AD30 group of Yuxi black pigs. These results suggested that an appropriate level of acorns in their diet could significantly improve the IMF content in the muscle tissues of Yuxi black pigs. Previous studies also found that free-range (acorns and grass) and low-protein diets significantly increased the IMF content in the muscle tissue (musculus serratus ventralis) of Iberian pigs [23]. Another study found that the IMF content of the *longissimus dorsi* of pigs fed acorns was higher than that of pigs fed acorns with *Curculio* sp. [35]. The IMF contents of the *longissimus dorsi* in this study were consistent with those found by previous researchers in Iberian pigs. These results indicated that Yuxi black pigs fed appropriate levels of acorns could also achieve a similar IMF content and meat quality as Iberian pigs. However, we found that excessively acorn-based diets (40%, 50%) significantly inhibited IMF deposition. This may be caused by differences in the nutrient tannin in acorns, which acts as an anti-trophic factor and is able to reduce feed intake and inhibit animal growth when a threshold is exceeded. But the specific reason needs further research.

### 4.3. Fatty Acid Composition

Growing research has found that the addition of acorns to feed improved the nutritional value of meat by regulating its fatty acid composition [21,36]. Previous studies demonstrated that the flavor substances provided by meat lipids (especially unsaturated phospholipids) are important factors affecting the eating quality of pork [37]. An excessive intake of SFA in humans will increase their plasma levels of low-density lipoprotein and cholesterol, which are related to an increased risk of atherosclerosis, coronary heart disease, and cardiovascular disease [29]. In this study, feeding with an acorn diet (AD30 group) significantly reduced the total SFA percentage in the *longissimus dorsi* and *biceps femoris* of Yuxi black pigs. A similar result was observed by a previous researcher, who found that the addition of acorns to the diet decreased the total SFA content in the *longissimus dorsi* of fatting pigs [21], which suggested that fatty acid composition was influenced by the addition of acorns. Additionally, Yuxi black pigs fed appropriate levels of acorns produced high-quality and healthy pork products (with a lower SFA content) for consumers.

In this study, the C18:1 percentage in the *longissimus dorsi* and *biceps femoris* of the AD30 group was higher than that of the control group, which indicated that feeding with an acorn diet could improve the MUFA composition in the muscular tissues of Yuxi black pigs. Similar results were found by a previous researcher, who reported that Iberian pigs fed with acorns and grass could had an increased C18:1 percentage in their subcutaneous fat, which demonstrated that the fatty acid composition of pig tissues was modified by dietary factors [23]. Another study reported that there was no difference in the percentage of C18:1 in perirenal fat between fattening rabbits fed with acorns and a control group. These results indicated that the effect of acorns on the MUFA percentage varies depending on animal species and tissues [38].

The n-3 fatty acids have been shown to reduce the risk of cardiovascular disease and promote brain development [39,40]. The n-6 fatty acids are mainly composed of linoleic acid (LA, C18:2 n-6) and arachidonic acid (ARA, C20:4 n-6). Previous studies have proven that LA has the effect of lowering blood pressure and preventing coronary artery disease [41], while arachidonic acid has the function of increasing the elasticity of blood vessels. Furthermore, PUFAs can also reduce plasma cholesterol and triglyceride levels and blood viscosity, thereby improving blood microcirculation and preventing the risk of heart disease [42]. Our results showed that the AD30 group had higher C18:2 n-6 and C18:3 n-3 percentages in its *longissimus dorsi* and C18:3 n-3 and C20:5 n-3 percentages in its *biceps femoris* compared with the control group, which suggested that appropriate levels of acorns in their diet could improve the fatty acid composition and meat quality of fattening pigs. These results indicated that pork from the AD30 group is beneficial to human health. However, another study found that the C18:2 n-6 and C18:3 n-3 percentages in the *longissimus dorsi* of Zlothnicka Spotted pigs fed with acorns were not affected. Inconsistent results in PUFA percentages may be caused by factors such as different feeding environments, pig breeds, feeding periods, and the amount of acorns in their diet.

### 4.4. Nutritional Indicators of Fatty Acid Composition

Dietary fats are fatty acids that may play a positive or negative role in the prevention and treatment of diseases. Different fatty acid compositions will affect the nutritional value of pork. PUFA/SFA is an indicator used to evaluate the impact of meat quality on cardiovascular health. The TI can assess the degree of thrombosis in many fatty acid composition studies; it shows the relationship between pre-thrombotic fatty acids and antithrombotic fatty acids, and eating foods with a lower TI can promote cardiovascular health [43]. A low cholesterol/hypercholesterolemia ratio (h/H) can reflect the effect of the fatty acid profile on cardiovascular diseases; it is also an important indicator in evaluating the ratio between unsaturated and saturated fatty acids [43].

In our study, we found that the values of PUSF/SFA and h/H were higher in the tissue of the *Longissimus Dorsal* and *biceps femoris* in the AD30 group compared to the control group, while the group’s TI values were lower, indicating that feeding pigs an acorn ration could improve the nutritional value of pork. Previous studies have found that the consumption of mulberry leaves rich in polyphenolic compounds could improve the fatty acid composition in adipose tissue and significantly increase PUFA/SFA values in high-fat-diet-induced obese mice [44]. Some studies showed that feeding grass carp an alfalfa diet could reduce their TI [45]. Other studies have shown that selected Polish mushrooms can improve the human h/H index, thereby preventing cardiovascular disease [46]. This study outcome is consistent with our results of improved pork fatty acid nutritional indicators after feeding pigs acorn diets. Therefore, the consumption of a diet containing a certain percentage of acorns can improve the meat quality of Yuxi black pigs.

### 4.5. Expression of Genes Involved in Lipid Metabolism

We hypothesize that there may be active substances in acorns that affect lipid metabolism and the differentiation of intramuscular adipocytes. The study of the expression of genes involved in the regulation of fat deposition is therefore of theoretical importance for the enhancement of intramuscular fat content. In this study, we found that genes involved in lipid synthesis and transcription factors promoting adipocyte differentiation were up-regulated and genes involved in lipolysis were down-regulated in the AD30 group after consuming an acorn diet. According to these results, it can be inferred that acorns have the potential to enhance the deposition of intramuscular fat by regulating the expression of genes associated with lipid metabolism.

The mechanisms of fat deposition are complex and are subject to a combination of enzymes and genes. *ACC* plays an important regulatory role in fatty acid synthesis; *ACC* is a biotin-dependent multidomain enzyme with biotin carboxylase and carboxylase activities in most eukaryotes. Firstly, biotin carboxylase catalyzes the ATP-dependent carboxylation of biotin using bicarbonate as its CO_2_ donor, while carboxytransferase promotes the transfer of carboxyl groups from biotin to acetyl-coa to form malonyl-coa. Malonyl-coa is an intermediate in the de novo synthesis of fatty acids and promotes acyl chain extension as a substrate for fatty acid synthase [47]. Increased *ACC* activity can increase the rate of adipogenesis [48]. *FAS* is the synthesis of long-chain fatty acids by combining acetyl coenzyme A with acyl carrier protein molecules and other auxiliary substances [49]. When *FAS* activity is enhanced or overexpressed, it can promote the deposition of triglycerides in the organism [50]. In this study, after consuming diets containing acorns, the expression level of *ACC* in the *biceps femoris* of black pigs in the AD30 group was significantly higher than that in the control group, as was the expression level of the *FAS* gene in their *longissimus dorsi* and *biceps femoris*. This study also found that *ACC* and *FAS* were significantly positively correlated with the *biceps femoris*. Canovas’s study showed that the intramuscular fat content of porcine semimembranosus was not correlated with the expression level of *ACC* [51]. Yang’s study found that the up-regulation of *ACC* gene expression promoted the accumulation of lipid droplets and synthesis of triglycerides, while the down-regulation of *ACC* gene expression reduced the accumulation of lipid droplets [52]. Similar to our results, an appropriate acorn diet could significantly up-regulate the gene expression levels of *ACC* and *FAS*, which could in turn improve IMF deposition, while an excessive acorn diet may inhibit IMF deposition. Cui found that *FAS* mRNA expression was correlated with the fat content in chicken liver, but there was no correlation between the IMF content in pectoral muscle and leg muscle tissue [53]. The differences in these results may be due to species or tissue-site differences.

*HSL* primarily catalyzes the second step of triacylglycerol degradation: the release of fatty acids from diacylglycerols [54]. In 2004, a study found an enzyme that preferentially hydrolyzes TAG and named it *ATGL* [55]. *ATGL* is mainly expressed in white and brown adipose tissue. [56]. *ATGL* is the enzyme that catalyzes the first step of the degradation of triacylglycerol to produce diacylglycerol and free fatty acids [57]. In our study, the expression level of *HSL* in the *longissimus dorsi* of Yuxi black pigs fed a diet containing acorns was significantly lower in the experimental group than in the control group. In the *biceps femoris*, the mRNA expression levels of *ATGL* and *HSL* in the AD30 group were significantly lower than those in the control group, while those in the AD50 group were significantly higher. This study also found that *ATGL* and *HSL* were significantly negatively correlated with the intramuscular fat content in the *biceps femoris*. Yang’s study found that an increased expression of *ATGL* in goat liver tissue led to a decrease in cellular lipids [52]. Liu’s study found that reduced *HSL* expression leads to increased IMF content and triglyceride content in pigs [58]. These results are consistent with our study; therefore, feeding pigs an appropriate acorn diet can down-regulate the expression of the *ATGL* and *HSL* genes in their *longissimus dorsi* and *biceps femoris*, thus promoting IMF deposition.

*PPARγ* plays an important role in maintaining the stability of the internal environment of animal lipid metabolism [59]. *PPARγ* mainly plays an important role in the regulation of cell proliferation and differentiation, glucose metabolism, and lipid biosynthesis. A variety of genes involved in fatty acid transport and metabolism are regulated at the transcriptional level by *PPARγ*, which can increase the expression of fatty acid transporters and fatty acid transport enzymes and stimulate the cellular uptake of fatty acids and their conversion to fatty acyl CoA [60]. *PPARγ* can activate *LPL*, *aP2*, *ACC,* and other genes related to lipid intake and adipogenesis in adipocytes, increase the synthesis of triglyceride, and promote the deposition of fat in adipocytes. *FABP4* is one of the genes highly expressed in animal adipose cells, and its protein is involved in long-chain fatty acid binding and plays an important role in fatty acid uptake, transport, and metabolism [61]. In this study, dietary acorn supplementation significantly increased the expression of the *PPARγ* and *FABP4* genes in the *longissimus dorsi* of black pigs in the AD20 and AD30 groups. It was also found that the expression of the *PPARγ* and *FABP4* genes in the *longissimus dorsi* and *biceps femoris* was significantly positively correlated with intramuscular fat content. Chen’s study found that the down-regulation of *PPARγ* expression leads to a decrease in adipogenic differentiation in pigs and thus inhibits fat generation [62]. Studies have shown that *FABP4* is related to intramuscular fat content [63]. In addition, Chen found that feeding an excessive energy diet promoted IMF deposition by increasing *FABP4* gene expression [64]. This is similar to our findings; these results indicate that dietary acorn supplementation can promote the expression of the *PPARγ* and *FABP4* genes in the *longissimus dorsi* and thus regulate IMF deposition.

In conclusion, a diet containing acorns may be involved in the synthesis, decomposition, and metabolism of fat, thus regulating the expression of lipid metabolism-related genes. The different gene expression levels in different muscular tissues may be due to the different mechanisms involved in lipid metabolism in these muscular tissues. However, how acorns regulate the expression of genes related to lipid metabolism remains to be further studied. In addition, the meat quality of black pigs fed with acorns is much higher than that of ordinary pigs. Although using acorns instead of corn will not save feed costs, the price of black pork is 3-4 times higher than that of ordinary pork in China, and the economic benefits are considerable. This will promote the utilization of acorns and provide a certain basis for the industrial development of acorns.

## 5. Conclusions

An acorn diet could regulate the IMF content, fatty acid composition, and lipid metabolism-related gene expression levels in the muscle tissue of Yuxi black pigs. It further affects the efficiency of fat deposition, thereby improving pork quality and providing a new theoretical basis for the development and utilization of new pig feed resources. After a comprehensive consideration of the results, we suggest that feeding pigs a 30% acorn diet had the best effect on producing quality pork products.

## Figures and Tables

**Table 1:** Composition and nutrition levels of the diets (air-dry basis).

Items	Content				
	CON	AD20	AD30	AD40	AD50
Ingredient, %					
Corn	65.5	51.3	44.9	38.7	32.1
Soybean meal	3.0	5.7	5.9	9.8	13.9
Wheat bran	22.2	14.3	7.5	3	0
Walnut dregs	5.3	4.7	7.7	4.5	0
Acorn	0	20	30	40	50
Premix	4	4	4	4	4
Total	100	100	100	100	100
Nutrient level					
Metabolic energy ME, MJ·kg^−1^	11.82	11.44	11.35	11.21	11.02
Crude protein CP, %	11.91	11.44	11.49	11.38	11.08
Lys, %	0.51	0.50	0.50	0.53	0.56
Cys, %	0.25	0.22	0.22	0.21	0.19
Met + Cys, %	0.47	0.41	0.41	0.40	0.36
Protein energy ratio, g·MJ^−1^	10.08	10.00	10.12	10.15	10.05

Premix ingredients (per kg of this product): Vitamin A: 120,000 IU; Vitamin D3: 45,000 IU; Vitamin E: 700 IU; Vitamin K3: 45 mg; Vitamin B2: 150 mg; Vitamin B6: 50 mg; Niacinamide: 750 mg; Calcium pantothenate: 460 mg; Choline chloride: 3.5 mg; Copper: 0.31 g; Iron: 3.5 g; Zinc: 1.4 g; Manganese: 0.8 g; Iodine: 42 mg; Selenium: 7.8 mg; Calcium: 16%; Phosphorus: 3.5%. CON: diet containing 0% acorns; AD20: diet containing 20% acorns; AD30: 30% acorn diet; AD40: 40% acorn diet; AD50: 50% acorn diet.

**Table 2:** Fatty acid composition of different acorn diet groups.

Percentage of Fatty Acids (%)	Content				
	CON	AD20	AD30	AD40	AD50
Myristic acid (C14:0)	0.1	0.27	0.35	0.43	0.52
Palmitic acid (C16:0)	12.27	13.08	13.08	13.63	14.36
Stearic acid (C18:0)	1.64	1.80	1.90	2.03	2.15
Oleic acid (C18:1)	23.53	24.96	25.73	26.70	27.61
Linoleic acid (C18:2 n-6)	56.88	55.64	55.32	54.42	53.41
Linolenic acid (C18:3 n-3)	2.83	2.90	3.04	2.93	2.79
UFA	83.35	83.25	83.49	83.22	82.84
SFA	14.28	13.91	13.55	13.56	13.63
U/S	5.84	5.98	6.16	6.14	6.08
ω-6	56.88	55.64	55.32	54.42	53.41
ω-3	2.83	2.90	3.04	2.93	2.79
ω-6/ω-3	20.10	19.19	18.20	18.57	19.14

**Table 3:** Primers used to analyze gene expression using real-time PCR.

Gene	GeneBank No.	Primer Sequence	Size, bp
*ACC*	AF175308.1	F:5′-CCTCTGCCTTCTGACATGCTGAC-3′	305
		R:5′-GCCAGTCCGATTCTTGCTCCAC-3′	
*ATGL*	EU373817.1	F:5′-GGGTCTGCCTGGGTGATACTGG-3′	374
		R:5′-GGTGATGGTGCTCTTGAGTTCGTAG-3′	
*FABP4*	NM_001002817.1	F:5′-AAGAAGTGGGAGTGGGCTTTGC-3′	320
		R:5′-AATTCTGGTAGCCGTGACACCTTTC-3′	
*FAS*	NM_213839.1	F:5′-CATCGTGAGGGTCAATTCTGCTGTC-3′	338
		R:5′-CATTTGGTGTTGCTGGTTGGTGTG-3′	
*HSL*	AF141958.1	F:5′-CTTTGCGGGTATTCGGGAACAGG-3′	212
		R:5′-TGTGGCTTGTGCGGAAGAAGATG-3′	
*PPARγ*	NM_214379.1	F:5′-GCAGGAGCAGAGCAAAGAGGTG-3′	345
		R:5′-GCCAGGTCGCTGTCATCTAATTCC-3′	
*GAPDH*	NM_001206359.1	F:5′-CAAGGCTGTGGGCAAGGTCATC-3′	279
		R:5′-AAGTGGTCGTTGAGGGCAATGC-3′	

*ACC*, acetyl-coa carboxylase; *FAS*, fatty acidsynthase; *HSL*, hormone-sensitive lipase; *ATGL*, adipose triacylglyceride lipase; *PPARγ*, peroxisome proliferator-activated receptor γ; *FABP4*, fatty acid transport protein 4, GAPDH, glyceraldehyde-3-phosphate dehydrogenase.

**Table 4:** Effect of acorn diets on fatty acid composition of *longissimus dorsi* of Yuxi black pigs.

Items	AD Level (%)					SEM	*p*
	CON	AD20	AD30	AD40	AD50		
SFA							
C10:0 (%)	0.083 ^ab^	0.093 ^a^	0.081 ^b^	0.079 ^b^	0.074 ^b^	0.003	0.001
C12:0 (%)	0.057	0.057	0.063	0.053	0.053	0.004	0.157
C14:0 (%)	1.283 ^ab^	1.337 ^a^	1.338 ^a^	1.283 ^ab^	1.237 ^b^	0.028	0.025
C15:0 (%)	0.046	0.047	0.042	0.064	0.051	0.006	0.058
C16:0 (%)	23.695 ^ab^	23.045 ^bc^	24.384 ^a^	22.584 ^c^	22.172 ^c^	0.383	0.001
C17:0 (%)	0.098 ^c^	0.133 ^b^	0.115 ^bc^	0.182 ^a^	0.106 ^bc^	0.013	0.001
C18:0 (%)	10.639 ^c^	11.681 ^b^	6.869 ^e^	9.325 ^d^	12.802 ^a^	0.280	0.001
C20:0 (%)	0.142 ^b^	0.203 ^b^	0.120 ^b^	0.532 ^a^	0.140 ^b^	0.006	0.001
C22:0 (%)	0.055 ^b^	0.094 ^a^	0.056 ^b^	0.064 ^b^	0.070 ^b^	0.008	0.008
C23:0 (%)	0.284 ^abc^	0.377 ^ab^	0.197 ^bc^	0.411 ^a^	0.263 ^c^	0.061	0.033
MUFA							
C14:1 (%)	0.036	0.036	0.024	0.025	0.033	0.010	0.557
C15:1 (%)	0.015	0.021	0.015	0.019	0.028	0.005	0.131
C16:1 (%)	5.995 ^a^	4.492 ^cd^	4.407 ^d^	5.116 ^bc^	5.665 ^ab^	0.302	0.001
C17:1 (%)	0.208 ^c^	0.203 ^c^	0.249 ^b^	0.284 ^a^	0.266 ^ab^	0.010	0.001
C18:1 (%)	47.855 ^b^	47.131 ^c^	52.322 ^a^	45.447 ^e^	46.429 ^d^	2.334	0.001
C20:1 (%)	0.892	1.097	0.958	0.871	1.018	0.093	0.174
C22:1 (%)	0.053	0.075	0.053	0.070	0.055	0.012	0.283
PUFA							
C18:2 n-6 (%)	6.665 ^e^	7.084 ^d^	7.138 ^c^	9.978 ^a^	7.379 ^b^	0.528	0.001
C18:3 n-6 (%)	0.023 ^bc^	0.036 ^a^	0.019 ^bc^	0.028 ^ab^	0.016 ^c^	0.004	0.008
C18:3 n-3 (%)	0.166 ^c^	0.205 ^b^	0.246 ^a^	0.268 ^a^	0.219 ^b^	0.011	0.001
C20:2 n-6 (%)	0.215 ^b^	0.246 ^ab^	0.271 ^a^	0.292 ^a^	0.247 ^ab^	0.020	0.033
C20:3 n-6 (%)	0.072 ^c^	0.117 ^ab^	0.060 ^c^	0.139 ^a^	0.092 ^bc^	0.016	0.003
C20:3 n-3 (%)	1.085 ^c^	1.758 ^b^	0.656 ^d^	2.404 ^a^	1.246 ^c^	0.142	0.001
C20:4 n-6 (%)	0.046	0.061	0.050	0.071	0.047	0.013	0.312
C20:5 n-3 (%)	0.046	0.066	0.059	0.073	0.050	0.010	0.128
C22:2 n-6 (%)	0.130	0.131	0.079	0.153	0.125	0.031	0.265
C22:6 n-3 (%)	0.095 ^b^	0.143 ^a^	0.113 ^ab^	0.151 ^a^	0.094 ^b^	0.018	0.028
Total SFA (%)	36.381 ^ab^	37.066 ^ab^	33.264 ^c^	34.576 ^bc^	36.970 ^a^	2.453	0.001
Total MUFA (%)	55.053 ^b^	53.054 ^d^	58.028 ^a^	51.832 ^c^	53.495 ^c^	2.360	0.001
Total PUFA (%)	8.542 ^b^	9.848 ^b^	8.693 ^b^	13.557 ^a^	9.515 ^b^	0.713	0.001

CON: diet containing 0% acorns; AD20: diet containing 20% acorns; AD30: 30% acorn diet; AD40: 40% acorn diet; AD50: 50% acorn diet. SEM, standard error of the mean. In the same row, the same lowercase letters indicate non-significant differences (*p* > 0.05), while different lowercase letters indicate significant differences (*p* < 0.05).

**Table 5:** Effect of acorn diets on fatty acid composition of the *biceps femoris* of Yuxi black pigs.

Items	AD Level (%)					SEM	*p*
	CON	AD20	AD30	AD40	AD50		
SFA							
C10:0 (%)	0.084 ^ab^	0.071 ^b^	0.080 ^ab^	0.090 ^a^	0.070 ^b^	0.006	0.034
C12:0 (%)	0.059 ^a^	0.044 ^b^	0.044 ^b^	0.047 ^b^	0.041 ^b^	0.005	0.022
C14:0 (%)	1.318 ^a^	1.162 ^b^	0.969 ^c^	1.277 ^ab^	1.176 ^b^	0.060	0.001
C15:0 (%)	0.120 ^a^	0.104 ^ab^	0.123 ^a^	0.083 ^b^	0.081 ^b^	0.010	0.004
C16:0 (%)	20.719 ^b^	20.095 ^c^	18.169 ^d^	22.266 ^a^	20.193 ^bc^	0.254	0.001
C17:0 (%)	0.203 ^b^	0.154 ^c^	0.142 ^d^	0.214 ^a^	0.141 ^d^	0.004	0.001
C18:0 (%)	8.787 ^a^	7.862 ^b^	5.303 ^d^	8.526 ^a^	5.969 ^c^	0.243	0.001
C20:0 (%)	0.097 ^a^	0.066 ^b^	0.045 ^c^	0.104 ^a^	0.093 ^a^	0.006	0.001
C22:0 (%)	0.166 ^a^	0.128 ^b^	0.188 ^a^	0.114 ^bc^	0.091 ^c^	0.013	0.001
C23:0 (%)	0.688	0.430	0.696	0.348	0.419	0.143	0.099
MUFA							
C14:1 (%)	0.056 ^b^	0.068 ^a^	0.069 ^a^	0.045 ^b^	0.044 ^b^	0.005	0.001
C15:1 (%)	0.084 ^a^	0.054 ^b^	0.088 ^a^	0.040 ^c^	0.043 ^bc^	0.005	0.001
C16:1 (%)	5.755 ^c^	5.910 ^c^	6.379 ^b^	6.825 ^a^	7.165 ^a^	0.169	0.001
C17:1 (%)	0.208 ^b^	0.237 ^b^	0.240 ^b^	0.306 ^a^	0.244 ^b^	0.017	0.002
C18:1 (%)	42.999 ^c^	47.283 ^b^	50.065 ^a^	47.337 ^b^	50.484 ^a^	0.457	0.001
C20:1 (%)	0.654 ^d^	0.728 ^c^	0.800 ^b^	0.840 ^ab^	0.873 ^a^	0.025	0.001
C22:1 (%)	0.192 ^ab^	0.162 ^b^	0.213 ^a^	0.079 ^c^	0.093 ^c^	0.014	0.001
PUFA							
C18:2 n-6 (%)	13.434 ^a^	11.485 ^b^	11.181 ^b^	8.672 ^d^	9.652 ^c^	0.168	0.001
C18:3 n-6 (%)	0.260 ^a^	0.248 ^a^	0.220 ^b^	0.252 ^a^	0.252 ^a^	0.005	0.001
C18:3 n-3 (%)	0.378 ^b^	0.296 ^c^	0.467 ^a^	0.264 ^c^	0.289 ^c^	0.022	0.001
C20:2 n-6 (%)	0.291 ^b^	0.285 ^bc^	0.258 ^c^	0.206 ^d^	0.333 ^a^	0.014	0.001
C20:3 n-6 (%)	0.176 ^b^	0.182 ^b^	0.225 ^a^	0.119 ^c^	0.140 ^c^	0.010	0.001
C20:3 n-3 (%)	2.332 ^b^	2.121 ^b^	2.958 ^a^	1.395 ^c^	1.542 ^c^	0.104	0.001
C20:4 n-6 (%)	0.163 ^ab^	0.135 ^b^	0.188 ^a^	0.095 ^c^	0.099 ^c^	0.013	0.001
C20:5 n-3 (%)	0.128 ^b^	0.109 ^bc^	0.180 ^a^	0.099 ^c^	0.089 ^c^	0.012	0.001
C22:2 n-6 (%)	0.389 ^b^	0.336 ^c^	0.440 ^a^	0.169 ^d^	0.195 ^d^	0.020	0.001
C22:6 n-3 (%)	0.260	0.246	0.269	0.188	0.186	0.032	0.068
Total SFA (%)	32.241 ^a^	30.115 ^b^	25.759 ^d^	33.068 ^a^	28.275 ^c^	0.429	0.001
Total MUFA (%)	49.948 ^e^	54.443 ^d^	57.854 ^b^	55.473 ^c^	58.946 ^a^	0.448	0.001
Total PUFA (%)	17.811 ^a^	15.442 ^c^	16.387 ^b^	11.459 ^e^	12.779 ^d^	0.148	0.001

CON: diet containing 0% acorns; AD20: diet containing 20% acorns; AD30: 30% acorn diet; AD40: 40% acorn diet; AD50: 50% acorn diet. SEM, standard error of the mean In the same row, the same lowercase letters indicate non-significant differences (*p* > 0.05), while different lowercase letters indicate significant differences (*p* < 0.05).

**Table 6:** Effects of acorn diets on the fatty acid constituent nutrient indexes in the *longissimus dorsi* of Yuxi black pigs.

Items	AD Level (%)					SEM	*p*
	CON	AD20	AD30	AD40	AD50		
n-6:n-3	5.078 ^b^	3.468 ^c^	7.019 ^a^	3.632 ^c^	4.862 ^b^	0.370	0.001
LA/ALA	40.241	34.561	29.019	37.246	33.819	3.273	0.059
PUFA: SFA	0.235 ^c^	0.267 ^b^	0.261 ^b^	0.392 ^a^	0.257 ^b^	0.014	0.001
EPA + DHA (%)	0.140 ^b^	0.209 ^a^	0.172 ^ab^	0.224 ^a^	0.144 ^b^	0.023	0.014
AI	0.453 ^a^	0.451 ^a^	0.446 ^ab^	0.424 ^c^	0.430 ^bc^	0.117	0.009
TI	2.995 ^a^	2.972 ^a^	2.963 ^b^	2.341 ^b^	6.231 ^a^	0.031	0.001
UI	74.161 ^b^	75.853 ^b^	77.172 ^b^	82.858 ^a^	74.778 ^b^	1.639	0.002
PI	12.340 ^c^	14.869 ^b^	12.395 ^c^	19.401 ^a^	13.515 ^bc^	0.969	0.001
HPI	3.150	3.029	3.188	3.222	3.374	0.116	0.128
NVI	2.456 ^b^	2.581 ^a^	2.428 ^b^	2.440 ^b^	2.657 ^a^	0.045	0.002
h/H	2.246 ^c^	2.365 ^b^	2.386 ^ab^	2.472 ^a^	2.390 ^ab^	0.204	0.002

AI, atherogenicity index; TI, thrombogenicity index; UI, fatty acid unsaturation index; PI, peroxidation trend index; HPI, Health-Promoting index; NVI, nutrition value index; h/H, hypocholesterolemic/hypercholesterolemic ratio. CON: diet containing 0% acorns; AD20: diet containing 20% acorns; AD30: 30% acorn diet; AD40: 40% acorn diet; AD50: 50% acorn diet. SEM, standard error of the mean. In the same row, the same lowercase letters indicate non-significant differences (*p* > 0.05), while different lowercase letters indicate significant differences (*p* < 0.05).

**Table 7:** Effects of acorn diets on the fatty acid constituent nutrient indexes in the *biceps femoris* of Yuxi black pigs.

Items	AD Level (%)					SEM	*p*
	CON	AD20	AD30	AD40	AD50		
n-6:n-3	4.752 ^ab^	4.574 ^b^	3.239 ^c^	4.891 ^ab^	5.067 ^a^	0.147	0.001
LA/ALA	35.753 ^ab^	38.807 ^a^	23.963 ^c^	33.012 ^b^	33.531 ^b^	1.897	0.001
PUFA:SFA	0.552 ^b^	0.513 ^c^	0.637 ^a^	0.347 ^e^	0.452 ^d^	0.010	0.001
EPA + DHA (%)	0.388 ^ab^	0.354 ^bc^	0.449 ^a^	0.287 ^c^	0.275 ^c^	0.036	0.003
AI	0.384 ^b^	0.354 ^c^	0.297 ^d^	0.409 ^a^	0.347 ^c^	0.008	0.001
TI	0.737 ^b^	0.692 ^c^	0.519 ^e^	0.832 ^a^	0.662 ^d^	0.013	0.001
UI	90.467 ^b^	89.751 ^b^	96.490 ^a^	81.659 ^d^	87.940 ^c^	0.453	0.001
PI	25.155 ^a^	22.317 ^b^	25.050 ^a^	16.971 ^d^	18.525 ^c^	0.281	0.001
HPI	2.608 ^c^	2.827 ^b^	3.370 ^a^	2.445 ^d^	2.882 ^b^	0.070	0.001
NVI	2.500 ^c^	2.745 ^b^	3.048 ^a^	2.509 ^c^	2.797 ^b^	0.054	0.001
h/H	2.760 ^c^	2.952 ^b^	3.474 ^a^	2.498 ^d^	2.961 ^b^	0.062	0.001

AI, atherogenicity index; TI, thrombogenicity index; UI, fatty acid unsaturation index; PI, peroxidation trend index; HPI, Health-Promoting index; NVI, nutrition value index; h/H, hypocholesterolemic/hypercholesterolemic ratio. CON: diet containing 0% acorns; AD20: diet containing 20% acorns; AD30: 30% acorn diet; AD40: 40% acorn diet; AD50: 50% acorn diet. SEM, standard error of the mean. In the same row, the same lowercase letters indicate non-significant differences (*p* > 0.05), while different lowercase letters indicate significant differences (*p* < 0.05).

**Table 8:** Correlation coefficients between lipid metabolism-related genes and IMF.

Gene	IMF Content			
	*longissimus dorsi*		*biceps femoris*	
	r	*p*	r	*p*
*ACC*	−0.147	0.601	0.819	0.001 **
*FAS*	0.077	0.784	0.812	0.001 **
*ATGL*	0.273	0.325	−0.653	0.008 **
*HSL*	−0.664	0.007 **	−0.870	0.001 **
*PPARγ*	0.940	0.001 **	0.741	0.002 **
*FABP4*	0.558	0.031 *	0.780	0.001 **

*ACC*, acetyl-coa carboxylase; *FAS*, fatty acidsynthase; *HSL*, hormone-sensitive lipase; *ATGL*, adipose triacylglyceride lipase; *PPARγ*, peroxisome proliferator-activated receptor γ; *FABP4*, fatty acid transport protein 4. IMF, intramuscular fat. Pearson correlation between IMF content and lipid metabolism genes is expressed as r. The data were statistically different at *p* < 0.05. One asterisk * indicates a significant difference (*p* < 0.05), and two asterisks ** indicate an extremely significant difference (*p* < 0.01).

## Data Availability

Data supporting the results of this study can be provided by the corresponding authors on reasonable demand.
